# Extensive Cortical Diffusion Restriction in a 50-Year-Old Female with Hyperammonemic Encephalopathy and Status Epilepticus

**DOI:** 10.1155/2014/257094

**Published:** 2014-04-24

**Authors:** Adam de Havenon, Kris French, Safdar Ansari

**Affiliations:** ^1^Department of Neurology, University of Washington, Box 359775, 325 Ninth Avenue, Seattle, WA 98104, USA; ^2^Department of Neurology, University of Utah, 50 North Medical Drive, Salt Lake City, UT 84132, USA

## Abstract

Comorbid hyperammonemic encephalopathy (HE) and status epilepticus (SE) leading to extensive cortical diffusion restriction (CDR) on MRI have not been previously reported. We describe a patient with HE who subsequently developed provoked SE. Sequential MRIs demonstrated a progressive CDR that involved the entire bilateral supratentorial cortex, thalami, and basal ganglia, resulting in death from cerebral edema and brain herniation. Diffuse CDR is most frequently seen after hypotension or hypoxia, which our patient did not experience. Such findings have also been described in both HE and SE (Milligan et al. (2009), Chatzikonstantinou et al. (2011), U-King-Im et al. (2011), and Bindu et al. (2009)), but not to the extent seen in our patient. Additionally, our patient had distinct radiologic features of both disease processes, suggesting a cumulative effect. The diagnosis of HE and SE in the setting of extensive CDR should not be missed and could lead to improved outcomes for two progressive, malignant, and treatable illnesses that can be easily overlooked.

## 1. Case Report

We present a 50-year-old female with cirrhosis secondary to chronic hepatitis C and alcohol abuse in remission who was admitted to our hospital with a three-day history of worsening abdominal pain and confusion. She was prescribed lactulose for the hepatic encephalopathy, which kept her ammonia in the normal range but had missed several doses over the preceding three days in addition to a decrease in the number of bowel movements. On initial exam the patient was normotensive and oxygenating well on room air with mild encephalopathy. A chronic ventral hernia displayed no signs of peritonitis. Labs on admission were significant for an ammonia level of 87 *μ*mol/L (18–72 *μ*mol/L), platelet level of 96 k/*μ*L, sodium of 132 mmol/L, and international normalized ratio (INR) of 1.6. Computed tomography of her abdomen and pelvis with intravenous contrast revealed that the hernia contained dilated small bowel but no definitive obstruction.

**Figure 1:**
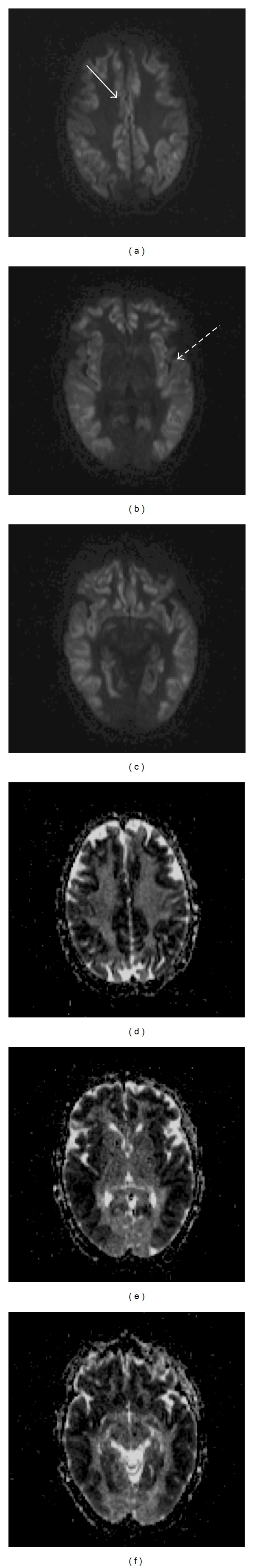
MRI of the brain six hours after the onset of SE. ((a), (b), and (c)) Axial diffusion tensor images (DTI) with extensive hyperintense cortical diffusion restriction, with the characteristic involvement of the cingulate gyrus (solid arrow) and insular cortex (spotted arrow) seen in HE; ((d), (e), and (f)) Axial apparent diffusion coefficient (ADC) images with corresponding hypointensity, characteristic of cytotoxic edema. The images are significant for the absence of diffusion restriction in the basal ganglia, thalamus, and relative sparing of the occipital lobes.

Overnight her mental status declined and by the next morning she was difficult to arouse and unable to follow commands. Vitals were within normal limits and morning labs were significant for a rise in plasma ammonia to 277 *μ*mol/L. She was electively intubated for airway protection but was not hypoxic. The following morning, on hospital day 3, she developed rhythmic clonic facial movements with eyelid fluttering, sustained tonic stiffening of the extremities, and intermittent right upper extremity jerks. The neurology service was consulted and continuous electroencephalogram (EEG) was recommended. During two hours of recording, prior to MRI imaging, there was focal status epilepticus in the left temporal region that spread to the left central and right temporal regions. 5 mg of intravenous lorazepam was administered and 1 gm levetiracetam twice daily was started, but the EEG continued to show persistent SE. She was paralyzed with vecuronium to obtain MRI of the brain with gadolinium contrast. This revealed extensive CDR involving the bilateral supratentorial cortex, with relative sparing of the occipital lobes, thalami, and deep gray matter. There was a corresponding decrease in apparent diffusion coefficient values, consistent with cytotoxic edema (Figure 1). Continuous neuromuscular blockade with intravenous cisatracurium was required to control tachypnea and burst suppression on EEG was achieved with high-dose propofol infusion.

**Figure 2:**
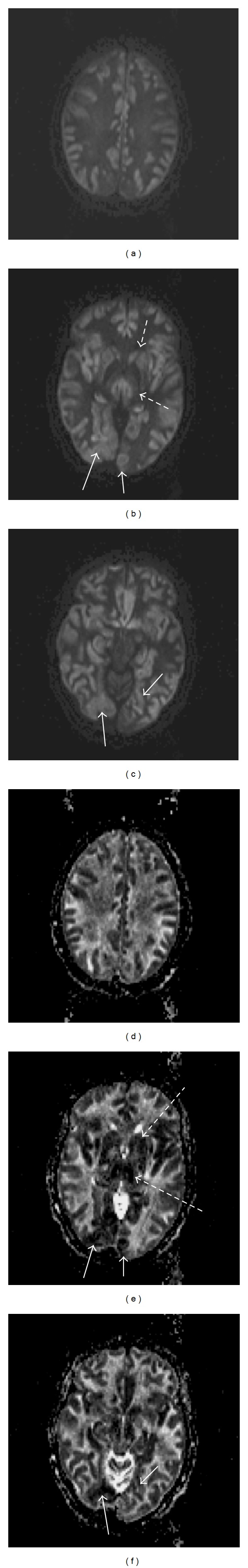
MRI of the brain four days after Figure 1. ((a), (b), and (c)) DTI images with hyperintense cortical diffusion restriction, now also involving ((b), dotted arrows) the thalami and basal ganglia, and ((b) and (c), solid arrows) the occipital cortices; ((d), (e), and (f)) axial apparent diffusion coefficient (ADC) images with hyperintensity of the previously hypointense cortical areas, confirming the transition from cytotoxic edema to extracellular edema, consistent with cerebral edema seen on CT. However, the areas of new diffusion restriction seen on the DTI have a hypointense ADC correlate ((e) and (f), corresponding solid and dotted arrows), suggesting they are due to a more recent event.

Over the next two days, on hospital days 4 and 5, the patient developed febrile sepsis but was not significantly hypotensive or hypoxic. On hospital day 6, despite the cessation of cisatracurium and propofol, she remained comatose with sluggishly reactive pupils and a weak gag response. EEG showed prolonged generalized suppression alternating with brief periods of low amplitude slow wave activity, ultimately progressing to diffuse attenuation. On hospital day 7, her exam remained unchanged and a repeat MRI brain demonstrated cerebral edema with bilateral uncal herniation and the extensive CDR seen on the first MRI, but there was spread of the CDR to the occipital cortices, the bilateral thalami, and basal ganglia. The prior areas of CDR were now bright on T2 and ADC, consistent with an evolution to extracellular cerebral edema, while the new areas of CDR were dark on ADC, suggesting a more recent insult still in the phase of cytotoxic edema (Figure 2). After discussion of the patient's prognosis with her husband, supportive care was withdrawn and the patient died shortly thereafter.

## 2. Discussion

Diffusion-weighted imaging (DWI) is most frequently used to identify the cytotoxic edema of ischemic stroke but can also help differentiate intracranial abscess, traumatic brain injury, and tumor [1]. Diffusion restriction on MRI usually involves both brain cortex and subcortex, often corresponding to a vascular territory after ischemic stroke or a localized area around a lesion, but some disease processes result in diffusion restriction that is specific to the cortex, such as hypoxic brain injury and hypoglycemia [2]. Cortical diffusion restriction (CDR) has been well described in patients in nonconvulsive SE and also postictally after SE [3]. Conventional T2 weighted MRI sequences often show hyperintensity, representing cerebral edema [4]. The diffusion restriction and edema associated with seizure can involve an entire cerebral hemisphere without respecting vascular territories or, in up to 15%, involve bilateral hemispheres, even in patients with a unilateral seizure focus on EEG [5–7]. The most common areas of MRI abnormality after SE are the temporal lobe, in particular the hippocampus, any lobe of the neocortex, and the thalamus. SE can result in massive cerebral edema, sometimes resulting in death [8].

The term hepatic encephalopathy refers to the neuropsychiatric dysfunction associated with liver dysfunction, while HE identifies the cause as elevated ammonia. The incidence of HE with extensive CDR is low, with only case reports in the published literature. HE has a high level of morbidity and mortality, with death rates as high as 50% in one case series. Radiographic abnormalities may be present even when the serum ammonia is less than 100 *μ*mol/L [9]. The diffusion restriction seen in HE is primarily cortical and frequently is diffuse and symmetric. The most common areas of diffusion restriction are the insular cortex and cingulate gyrus, which are involved in almost all patients. It is rare to have involvement of the occipital cortices in HE, and involvement of the thalami and basal ganglia has only been reported in one patient [2, 10]. In addition to cortical diffusion restriction there is often generalized cerebral edema, which can lead to death [9]. Seizure is a common complication of HE, presumably the result of cytotoxic edema [9].

## 3. Conclusion

Based on the clinical and radiologic abnormalities, the patient's extensive CDR was most likely the result of both HE and SE in a sequential manner. There was no evidence for other etiologies of extensive CDR, in particular no documented hypotension, hypoxia, or hypoglycemia. The distribution of diffusion restriction on the initial MRI is most consistent with published descriptions of HE. Furthermore, the MRI was only hours after the onset of SE, while the patient's ammonia level had been increasing over days, allowing for more opportunity to create such an abnormal MRI. The diffusion restriction was almost all cortical and symmetric; it spared the occipital lobes and, more importantly, the thalami and basal ganglia, which have only been involved in one case report of HE [2, 10].

On the subsequent MRI, 4 days later, there was new CDR in the occipital lobes, thalami, and basal ganglia, while the original areas of CDR had progressed to cerebral edema. Involvement of the thalamus is of particular importance because it is common in SE and not in HE. The left hemispheric predominance of epileptiform activity on EEG during SE may suggest that the MRI would be unilaterally abnormal, but the patient had periods of spread to the right hemisphere on the initial EEG and was off EEG while paralyzed for at least 7 hours during the episode of SE when she may have had further generalization. Furthermore, bilateral CDR has been described after focal SE on EEG [5]. The HE provoked the seizure, as no other causes for a provoked seizure were identified and she had no history of epilepsy.

Comorbid HE and SE leading to cumulative CDR have not been previously reported and the extent of CDR in this case was exceptional. Our case highlights that whenever extensive CDR is present, one should consider hypoxic and hypoglycemic insult first as they are common and characteristic of this pattern of injury but also consider HE and SE [2], which necessitate diagnostic tests that may not otherwise be performed. In this clinical setting, a serum ammonia level should be checked and trended over at least 2 days. If it is elevated and sufficient ammonia elimination cannot be achieved through the gastrointestinal tract, other methods should be considered such as hemodialysis, rifaximin, or branched-chain amino acids [11]. While convulsive SE is a clinical diagnosis, these findings on MRI warrant an EEG to identify nonconvulsive status epilepticus. Aggressive treatment of SE, even medically-induced coma in refractory cases, is necessary because morbidity and mortality are directly affected by the duration [12]. The diagnosis of HE or SE in the setting of extensive CDR should not be missed and could lead to improved outcomes for two progressive, malignant, and treatable illnesses.
